# Bright and ultrafast electron point source made of LaB_6_ nanotip†

**DOI:** 10.1039/d3na00069a

**Published:** 2023-03-24

**Authors:** O. Bhorade, B. Deconihout, I. Blum, S. Moldovan, J. Houard, A. Normand, K. Jagtap, M. More, A. Vella

**Affiliations:** a Univ. Rouen Normandie, INSA Rouen Normandie, CNRS, Groupe de Physique des Matériaux Avenue de l'Université BP 12 76801 Saint Etienne du Rouvray France angela.vella@univ-rouen.fr +33 232 955054 +33 232 955168; b Department of Physics, Savitribai Phule Pune University Pune 411007 India

## Abstract

The development of time-resolved transmission electron microscopy (TEM), ultrafast electron spectroscopy and pulsed X-ray sources relies on the realization of stable and high brightness sources of ultra-short electron bunches with a long service time. The flat photocathodes implanted in thermionic electron guns have been replaced by Schottky-type or cold-field emission sources driven by ultra-fast laser. Recently, lanthanum hexaboride (LaB_6_) nanoneedles have been reported to have high brightness and high emission stability when working in a continuous emission mode. Here, we prepare nano-field emitters from bulk LaB_6_ and we report on their use as ultra-fast electron sources. Using a high repetition rate laser in the infrared range, we present different field emission regimes as a function of the extraction voltage and laser intensity. The properties of the electron source (brightness, stability, energy spectrum and emission pattern) are determined for the different regimes. Our results show that LaB_6_ nanoneedles can be used as ultrafast and ultra-bright sources for time-resolved TEM, with better performances as compared to metallic ultra-fast field-emitters.

## Abstract 2

For the first time, we investigate strong-field photoelectron emission from LaB_6_ needles, opening the way towards new sources of ultra-short pulses of electrons.

## Graphical abstract

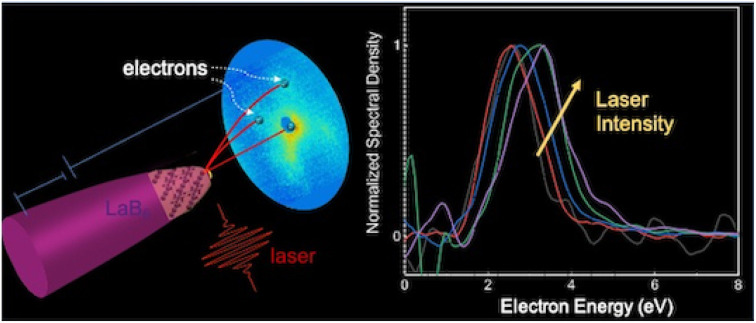

## 1 Introduction

Ultrashort electron beams are used for a wide range of applications such as electron microscopes^1–4^ and portable X-ray sources.^5,6^ In addition, electron laser emission from nanoneedles is also an interesting system for the study of strong field phenomena in materials at the nanoscale.^7–10^

The control of the electron emission by ultra-fast laser was extensively studied in the last ten years, starting from flat photocathodes to Schottky-type electron sources or cold-field emission sources. These ultra-fast field emitters have also been integrated as electron guns in ultra-fast electron microscopes.^11,12^ So far, the best performances for high resolution scanning transmission electron microscopy (STEM) are obtained using tungsten sources. They present however a significant decay of the emission current in less than one hour (in static and ultra-fast emission regime) and stochastic current fluctuations. Many studies have been reported presenting different strategy to bypass these limitations, *e.g.* the use of new materials such as carbides, borides carbon nanotubes or diamond nano-cones.^13–16^

Lanthanum hexaboride (LaB_6_) has been extensively explored over the past decades because of its successful application as a thermionic electron source due to its strong mechanical stability, its low work function and its high melting point.^17^ However, little success has been reported up to recent works on single LaB_6_ nanowires demonstrating that LaB_6_ is a promising material for cold field emitters.^18–22^ Since nanowires are too fragile and complicated to manipulate, last year S. Tang *et al.*^23,24^ proposed the use of LaB_6_ nanoneedles as robust, ultra-bright emission sources, with a long time emission stability.

We report herein on the fabrication of nano field emitters from bulk LaB_6_ and on their use as cold field emitter sources that can operate in continuous (DC) and ultra-fast emission mode with a stable emission during several hours, in both modes. We also demonstrate that, by changing the extraction voltage and laser intensity, the emission properties can be controlled, in terms of emission pattern, energy spectrum and current. This work opens the way towards the development of new sources of ultra-short pulses of electrons based on LaB_6_ nanotips.

## 2 Experimental

### 2.1 Fabrication of the LaB_6_ electron source and its structural characterization

**Fig. 1:**
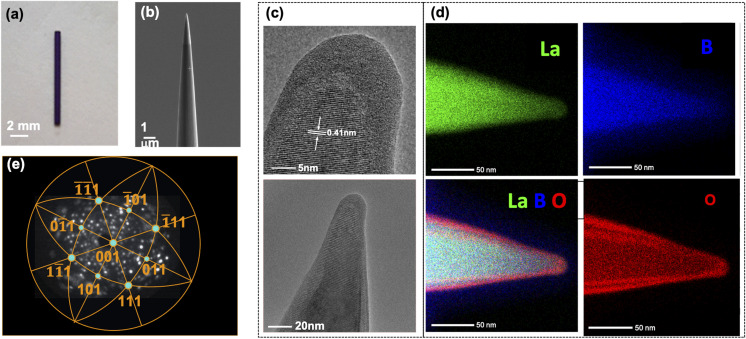
(a) Optical microscopy image of the LaB_6_ rod. (b) Scanning electron microscopy (SEM) image of a LaB_6_ nanoneedle as prepared. (c) High resolution transmission electron microscopy (HRTEM) image of the nanoneedle, showing the radius of curvature of 10 nm and the interplanar distance of 0.41 nm. (d) EDX map of the chemical composition and distribution of lanthanum (La), boron (B), oxygen (O) and the La + B + O. (e) FIM pattern of the tip in H_2_ gas and stereographic projection showing the crystallographic orientations of the tip's hemispherical surface.

We used a highly reproducible two-step method to fabricate the LaB_6_ nanotips. The bulk LaB_6_ single crystal rods of 0.7 mm in diameter and 10 mm in length and oriented according to the 〈100〉 direction were purchased directly from the company A-P Tech, USA (see Fig. 1(a)). The rod was then cramped in a nickel capillary of 0.8 mm inner diameter. The tip of the LaB_6_ rod was electrochemically etched in a 10% HNO_3_ solution to form a sharp apex.^19^ A Focused Ion Beam Scanning Electron Microscopy (FIB-SEM) dual beam system (Helios 5 Plasma FIB) using Xe ions was used to further sharpen the tip to an apex diameter of less than 20 nm. Most of the milling was performed using 30 keV ions, while 12 keV ions were used in the final milling step in order to minimize the presence of defects in the final sample. Note that our method is more robust than the method proposed in the previous work on LaB_6_ nanoneedles,^18,23^ because in our case the entire needle is made of LaB_6_, hence we avoid having to form a bond with the tungsten filament which can compromise mechanical stability as well as electrical and thermal conductivity.

### 2.2 Structural characterization

The structure and chemical composition of the LaB_6_ nanotip were characterized using a double corrected analytical TEM (JEOL ARM) operated at 200 kV and equipped with a Centurio 100 mm^2^ X-rays spectrometer dedicated to the Energy Dispersive X-rays Spectroscopy (EDS) and an Electron Energy Loss Spectrometer (EELS). Fig. 1(c) displays the high-resolution TEM image of the apex after its preparation by FIB. A lattice spacing of 0.41 nm was measured, which corresponds to the (100) crystallographic orientation of the LaB_6_ crystal as known from the literature.^25^ No other crystal orientation was identified along the tip structure. The micrograph also shows the presence of an amorphous layer on the surface, with a thickness of about 5 nm. The chemical composition of the tip was further explored by Energy Dispersive X-ray Spectroscopy (EDS) for mapping the constitutive chemical elements. The predominant signals of lanthanum and boron arise from the tip whereas the oxygen is detected within a surface oxide layer. The xenon implantation cannot be studied as the corresponding Xe peak overlaps with the La signal. The image of the overlapped maps corresponding to the B, La and O (Fig. 1(d)) reveals an O concentration gradient with a lower concentration in the bulk. During this investigation, a Fischione 360° rotation holder adapted to the use of sharp tips was employed for both the imaging and the EDS experiments. The HAADF micrographs (1024 × 1024 px) were registered using 20 ms px^−1^, whereas the EDS maps (256 × 256 px) were acquired for 20 minutes and using a 0.02 ms px^−1^. For the EDS mapping, a spatial drift correction was applied every 60 seconds.

### 2.3 Field ion microscopy (FIM)

The field ion microscopy (FIM) was done in an ultra-high vacuum stainless steel chamber maintained at a base pressure of 1 × 10^−10^ mbar. A positive voltage was applied to the nanotip. The FIM pattern was obtained by cooling the LaB_6_ nanotip to 77 K using liquid N_2_ and introducing H_2_ as imaging gas at a partial pressure of 1 × 10^−5^ mbar. FIM was performed in order to remove the surface oxide layer in a controlled way and ensure a clean tip surface. A positive voltage of 8 kV was applied to remove the oxide layer by field evaporation. Then, the FIM pattern was recorded at a positive voltage of 7.5 kV. Fig. 1(e) shows the crystallographic structure of the LaB_6_ sample, with stereographic poles arranged in a pattern corresponding to a cubic structure. The large (001) pole is seen at the center of the detector. The other (011) and (111) poles and their equivalent poles can be seen symmetrically aligned around the (001) pole.

### 2.4 Field electron emission and laser-assisted electron emission measurements

Field emission (FE) measurements were performed in an ultrahigh vacuum system at a pressure of 4 × 10^−10^ mbar at room temperature. A negative bias voltage *V*_tip_ is applied to the LaB_6_ tip. The electron kinetic energy spectrum is measured with a retarding field energy analyser using concentric hemispherical grids. The kinetic energy of the emitted electrons can be measured with a resolution of about 0.2% of *V*_tip_. The detector is composed of a stack of two micro channel plates (MCP) coupled with a phosphorescent screen each of 8 cm in diameter with the first MCP located at a distance of about 6.5 cm from the tip. This setup enables the measurement of the 2D map of the emission pattern. The MCPs have a hole in the center of 0.5 cm in diameter. A CCD camera is used to recorded the impacts on the phosphorescent screen. The detected current is then calculated using the measured number of spots per recorded image. The CCD camera's exposure time can be varied from 20 μs to several seconds.

Due to the limitation in the number of spots that can be discriminated on each image, measurable currents are limited to tens of fA. In order to go up to the pA range, a pico-ammeter is also connected to the phosphorescent screen in order to measure the current generated by electron impacts on the screen after amplification by the MCPs. This output current is directly correlated to the image intensity measured on the camera as well as the number of counted impacts, at low intensity.

We use a femtosecond fiber laser emitting at a wavelength of 2.25 μm, with transform limited pulses with 130 fs pulse duration and an output power of 300 mW at a repetition rate of 13 MHz, from the company NOVAE. The laser beam is focused on the tip emitter through a spherical mirror of numerical aperture NA = 0.3 and a working distance WD = 25 mm. The beam waist at the focal point is 14 μm (diameter at 1/*e*^2^). The alignment of the laser on the sample is checked using the diffraction seen by a long-distance microscope objective on a CCD camera mounted perpendicular to the laser propagation direction. Confirmation of the exact alignment is corroborated by rotating the polarization of the laser light. As stated by Barwick *et al.*,^8^ when correctly aligned, electron emission is negligible for a polarization perpendicular to the tip axis, as presented in the ESI.†

## 3 Results and discussions

### 3.1 Field emission characteristics

**Fig. 2:**
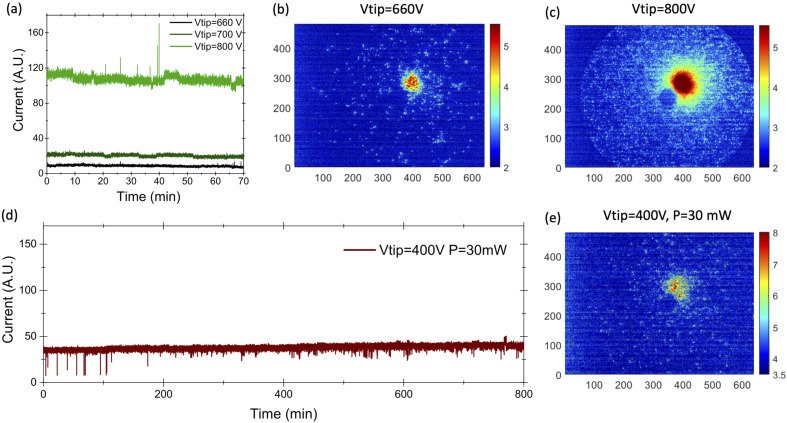
(a) Short term stability for three values of the extracting voltage. (b) FEM patters for DC field emission at *V* = 600 V; (c) FEM pattern for DC field emission at *V* = 800 V; (d) long-term stability for multiphoton photoemission at the extraction voltage of 400 V and laser intensity of 2.8 × 10^10^ W cm^−2^. (e) FEM pattern, for the emission reported in (d).

Prior to field electron emission experiments, the samples are activated by applying a high positive voltage of several kV (about 7–8 kV) which removes the occasional surface contamination after FIM imaging. These activation procedure allows us to obtain a single emission spot, as reported in Fig. 2(b and c).

Afterwards, a negative voltage supply was connected to the emitter and when increasing the voltage a field electron microscopy (FEM) pattern is obtained (Fig. 2(b)). By comparing this pattern with the FIM pattern, it is shown that the electron emission site corresponds to the (001) crystallographic pole (Fig. 1(e)). By fitting the emission spot with a Gaussian function, a full width at half maximum (FWHM) of 4 mm is obtained, which corresponds to a divergence semi-angle of 0.03 rad (see Fig. 2(b)). For an emission current of 0.5 pA at 660 V, a brightness of about 10^9^ A m^−2^ sr^−1^ is calculated in a first approximation by the estimation of the physical source size (more detail in the ESI†). This value is lower than the one reported in the recent works on the emission from LaB_6_. However, by increasing the applied voltage from 600 V to 800 V, the solid angle of divergence increases up to 0.05 rad (as shown in Fig. 2(c)) and then stays almost stable whilst the emitted current increases from 0.5 pA to 10 nA. Then a brightness of 10^12^ A m^−2^ sr^−1^ close to the values reported in ref. 23 and 24 of (7–12) × 10^12^ A m^−2^ sr^−1^ is obtained.

We studied the short-term emission stability in dark (DC mode) over 1 h 10 min without any cleaning. The current stays stable and the amplitude of the high frequency current fluctuation (hereafter called tip noise) has a relative standard deviation of 10% at low voltage and it decreases to 3.5% at high voltage. These values are higher than the 1% reported for cold field emitter and recently for LaB_6_ nanoneedles.^20,23^ Tip noise generally arises from the absorption/desorption processes, surface diffusion of absorbed molecules. It therefore depends on the vacuum level around the tip that in our experimental set-up is 10 times higher than in standard TEM ultra high vacuum chamber.^26^

By illuminating the tip with the 2.25 μm laser the emission of electrons occurs at lower extraction voltages. We studied the long-term emission stability under illumination at a power of 40 mW over 13 hours without any cleaning. The current remains stable and the amplitude of the high frequency current fluctuation increases from 4% (during the first 2 hours) up to 7.5% over the last 2 hours. These values are in the same range as the value we measured in DC emission and therefore we can state that the laser illumination does not affect the tip noise or the long-term stability.

The FEM pattern shows that the emission still originates from the (001) pole but the diameter of the emission pattern is larger, with a FWHM of 7 mm, corresponding to a divergence semi-angle of 0.05 rad. From FIM and FEM patterns we estimated a radius of the source of 0.43 nm (more detail are presented in the ESI†). The measured value of the current was 250 fA, which corresponds to a brightness of 4.5 × 10^7^ A m^−2^ sr^−1^. This value of the brightness is close to the value of 2.2 × 10^7^ A m^−2^ sr^−1^ reported in ref. 11 for laser-driven cold field emission from a tungsten tip. The electron emission occurs during 130 fs, every 77 ns, related to the repetition rate of the laser of 13 MHz. Assuming that the emission between laser pulses is negligible, these values give an instant brightness of 3 × 10^13^ A m^−2^ sr^−1^ during laser pulses, which is similar to the values measured in continuous DC emission for state-of-the-art cold field emitters.^11,26^

**Fig. 3:**
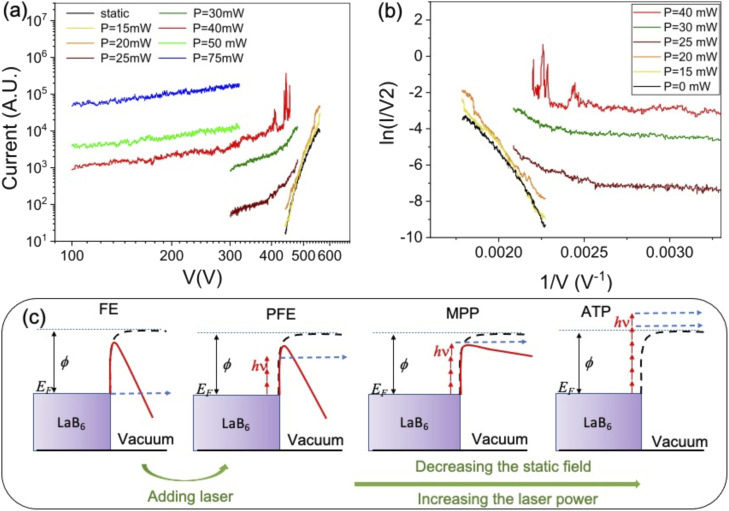
(a) Current–voltage characteristic of electron emission obtained from an LaB_6_ needle: in dark (black line) and under laser illumination at 2.25 μm, increasing the intensity from 3 GW cm^−2^ (red) to 15 GW cm^−2^. (c) The Fowler–Nordheim representation of the data in (a) for a laser intensity from 3 GW cm^−2^ (red) to 8 GW cm^−2^. (c) Electron emission processes: (FE) DC emission or field emission: the quantum tunneling of electrons through an electric-field induced narrow potential barrier into vacuum. *E*_F_ Fermi level, (PFE) photoassisted field emission: an electron is excited to an intermediate state of higher energy by photon absorption, and then tunnels through the barrier; (MPP) multiphoton photoemission: an electron absorbs the energy of several photons and overcomes the barrier for photoemission; and (ATP) above threshold photoemission: the electron absorbs the energy of more than the minimum number of photons required to overcome the barrier for photoemission, even in the absence of an electric field.

The relationship between the emission current and the extraction voltage follows Fowler–Nordheim's (FN) equation:1
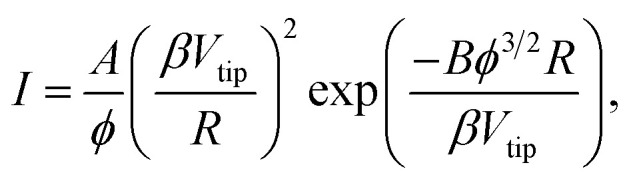
derived for metallic emitters, where *β* is the field enhancement factor, *R* the end radius of the needle of 10 nm, *A* = 1.56 × 10^−6^ A V^−2^, *B* = 6.83 × 10^9^ eV^−3/2^ V m^−1^ and *ϕ* is the work function.^27^ The (*I*, *V*) characteristic of LaB_6_ samples is reported in Fig. 3(a) and (b), in log–log scale and in FN coordinates, respectively.

**Fig. 4:**
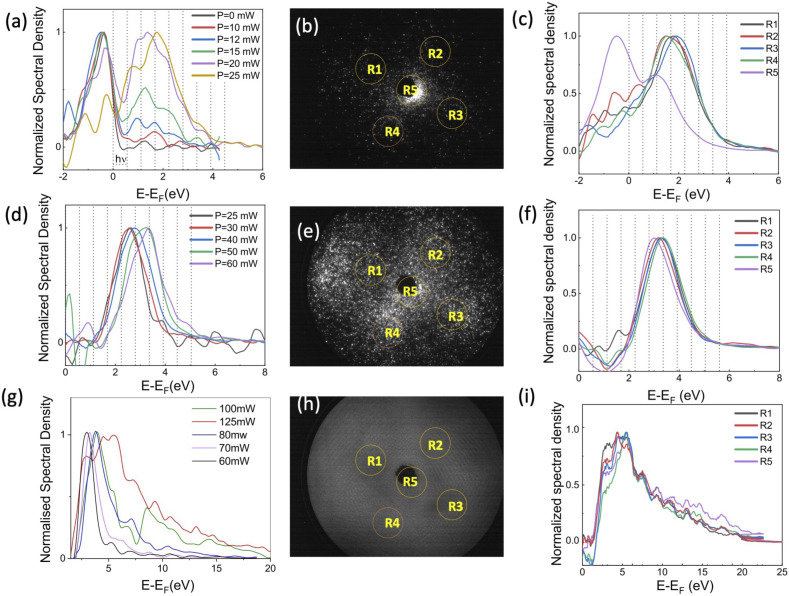
(a) Energy spectra for *V* = 570 V in static emission mode (black line) and under laser illumination varying the intensity from 2 GW cm^−2^ (red) to 5 GW cm^−2^ (orange). (b) FEM pattern at *V* = 570 V and *I* = 2 GW cm^−2^. (c) Energy spectra for each area indicated by a red circle in (b) at *V* = 570 V and *I* = 2 GW cm^−2^. (d) Energy spectra for *V* = 300 V under laser illumination varying the intensity from 5 GW cm^−2^ (black) to 12 GW cm^−2^ (purple). (e) FEM pattern at *V* = 300 V and *I* = 10 GW cm^−2^. (f) Energy spectra for each area indicated by a red circle in (e) at *V* = 300 V and *I* = 10 GW cm^−2^. (g) Energy spectra for *V* = 100 V under laser illumination varying the intensity from 12 GW cm^−2^ (black) to 25 GW cm^−2^ (red). (h) FEM pattern at *V* = 100 V and *I* = 25 GW cm^−2^. (i) Energy spectra for each area indicated by a red circle in (h) at *V* = 100 V and *I* = 16 GW cm^−2^.

The illumination of the cathode by 0.55 eV photons leads to a substantial increase in the emission current, as observed in Fig. 3(a), where the (*I*, *V*) characteristics are reported for different values of the laser intensity and the extraction voltage; for example, for an applied voltage of 450 V and a laser intensity of 5 or 6 GW cm^−2^ (power of 25 or 30 mW), the current is multiplied by a factor of 10 or 100. Moreover, at 300 V increasing the laser intensity from 5 to 15 GW cm^−2^ (from 25 to 75 mW) leads to the current augmentation by more than a factor 1000. At the same time the emission pattern changes, as shown in Fig. 4(e). However, the brightness remains high, of about 5, 5 × 10^6^ A m^−2^ sr^−1^ at 13 MHz and in the corresponding DC mode 3.7 × 10^13^ A m^−2^ sr^−1^, for a divergence semi-angle of 0.5 rad.

Under laser illumination, the *I*–*V* curves show a linear dependence (in FN coordinates) at high voltages and a constant behavior at low voltages and high laser power, as shown in Fig. 3(b). Therefore, different emission regimes can be identified from the FN plots: the photo-field emission (FE or photo-assisted field emission) at high voltages and low laser power and the multiphoton photoemission (MPP), at low voltages and higher laser power, as introduced in previous works on metallic field emitter^7,28^ and represented by a schematic in Fig. 3(c). For voltages lower than 300 V and laser powers higher than 40 mW (8 GW cm^−2^), the (*I*, *V*) plots show a linear behavior (see Fig. 3(a)), as expected for above-threshold photoemission (ATP).^29^ Moreover the emitted current scales as *P*_laser_^*n*^ where *n* = 3.6 indicates the mean number of photons involved in the ATP process (data available in the ESI†).

The *I*, *V* characteristics of Fig. 3(a) show a high nonlinearity at low laser intensity and high voltages, according to photo-assisted FE. By increasing the laser power and reducing the voltages the nonlinearity strongly decreases according to the MPP process.

### 3.2 Electron spectroscopy

The electron energy measurements were performed using a retarding field energy analyzer. To study the photoassisted field emission, the tip voltage was set to *V*_tip_ = 570 V. The photoelectron spectra are shown in Fig. 4(a) for laser intensities ranging from 2 GW cm^−2^ to 5 GW cm^−2^. These spectra are obtained using a scanning resolution of 0.1 V over 100 ms per step and with a Savitzky–Golay smoothing filter on 7 adjacent points. The spectra are normalized at their maximum intensity to focus on their spectral shape. The low kinetic energy peak corresponds to the emission of electrons from the Fermi level, as shown in Fig. 3(c) for FE process. The second peak at higher energy corresponds to the photoassisted field emission (PFE) by the absorption of 1, 2, 3 or 4 photons. This peak is obtained by the convolution of the different peaks related to single or multi photon absorption. The energy resolution of the spectrometer at 570 V is of 1.2 eV, therefore the contribution of the 1, 2, 3 or 4 photons can not be discerned. As the laser intensity increases, the amplitude of the second peak increases, indicating that the photoassisted field emission becomes more favorable, as already reported on W field-emitters.^30,31^

The FEM pattern shows that the main emission is still from (001) pole. However, electrons are also emitted from the (011) poles and a four-fold symmetry is visible on the FEM pattern. Thanks to the 2D detector, the detected current can be integrated in a reduced area in order to extract the spectrum corresponding to each emission site. The five selected areas on the detector system, corresponding to the (001) and the four (011) poles, are indicated by yellow circles in Fig. 4(b). The energy spectrum from the (001) pole, area R5, differs from the spectra of the (011) poles (see Fig. 4(c)), with a stronger emission from the Fermi level and a maximum of the photoassisted field emission peak at 1 eV, indicating an emission after the absorption of 2 photons. The emission from the (011) poles is mainly due to the PFE mechanism with a peak maximum at 1.5 eV, associated with the absorption of 3 photons. The work function corresponding to the (100) poles in LaB_6_ nanoneedles was measured at 2.7 eV, close to the 2.6 eV reported on flat surfaces.^24,25^ The reduction in the peak corresponding to emission from the Fermi level, on the (011) poles, indicates that the (011) work function is higher than 2.7 eV. Therefore, after the absorption of 2, 3 photons the tunneling barrier that the electrons have to pass in order to be emitted from (011) poles is larger than that of the (001) pole. This can explain the shift of the muti-photonic peak from 1 eV (∼2 photon) for (001) pole, to 1.5 eV (∼3 photons) for (011) poles.

In order to study the multiphotonic photoemission (MPP), the tip voltage was reduced to 300 V. Fig. 4(d) shows the energy spectra for laser intensities ranging from 5 GW cm^−2^ (*P* = 25 mW) to 12 GW cm^−2^ (*P* = 60 mW). No emission has been reported at kinetic energy equal to the Fermi energy, however a peak is visible around 2.5 eV and it shifts towards higher energies increasing the laser intensity. The contribution of different multiphotonic absorption can not be separated because electrons come from regions with different work functions and the spectrometer resolution at 300 V is of 0.6 eV. At lower laser intensity the peak corresponds to the 4–5 photons MPP. The increase the number of photons involved, compared to the case of an extraction voltage of 570 V, is due to the increase on the effective work function (from 1.5 to 1.8 eV, as visible from low-energy cut-off spectra) and the increase of the contribution of the emission from the (011) poles.

Starting with a laser intensity of 10 GW cm^−2^ the maximum of the spectrum shifts towards high energy with about 0.5 eV when increasing the laser intensity. This effect is most probably due to ponderomotive effect and it reflects that a photoelectron must oscillate in the optical field after photoemission. In strong fields regime, its average kinetic energy, *e.g.* the ponderomotive energy *U*_p_, is not negligible. Therefore, if a transition takes place from an initial state to a final state, the average kinetic energy *U*_p_ must be provided in addition to the energy difference. This is equivalent to an increase of the work function by *U*_p_.^32,33^ When *U*_p_ is equal to the photon energy, the lowest energy emission channel is suppressed. The ponderomotive energy is equal to:2
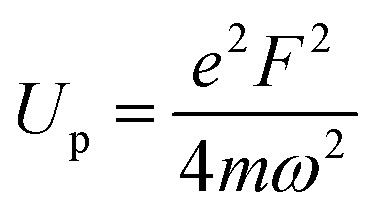
with *e* and *m* the charge and mass of the electron, *ω* the laser frequency and *F* the local electric field of the laser given by:3
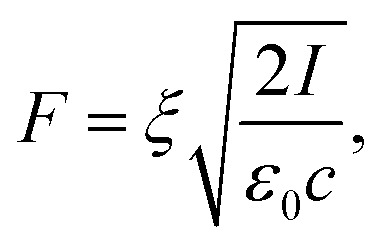
where *ξ* is the field enhancement, *c* the speed of the light in vacuum and *ε*_0_ the vacuum permittivity. For *I* = 10 GW cm^−2^, we can consider *U*_p_ = ℏ*ω* and from eqn (2) and (3) we can calculate the value of the enhancement factor *ξ* = 11.

As shown in Fig. 4(e), at high laser intensity the FEM pattern shows a four-fold symmetry with an emission from the (011) poles of almost the same intensity as the (001) pole. The energy spectra from the 5 emission areas don't show any significant difference. Because as the electrons are ejected above the barrier, as shown in Fig. 3(c) for MPP emission, the difference in the work function between the (001) and (011) poles does not matter anymore. At low laser intensity, the emission pattern shown in the ESI† is similar to the FEM image obtained at high voltage. In this case the emission from the central pole is the most intense and the energy spectra from the (011) poles are shifted towards higher energies. Here, the difference in work function between the (001) and (011) poles plays a role again. The spectra are shown in the ESI.†

The decrease of the applied voltage from 570 V to 300 V with the associated increase of the laser intensity from 4 to 10 GW cm^−2^ induces an increase of the number of photons involved in the emission process going from (2–3) to a (5–6) photon process. This increase of the nonlinearity is mainly associated to the increase of the laser power when the applied voltage is reduced. The nonlinearity of the process is higher for the emission from the 110 poles due to their higher work function.

**Fig. 5:**
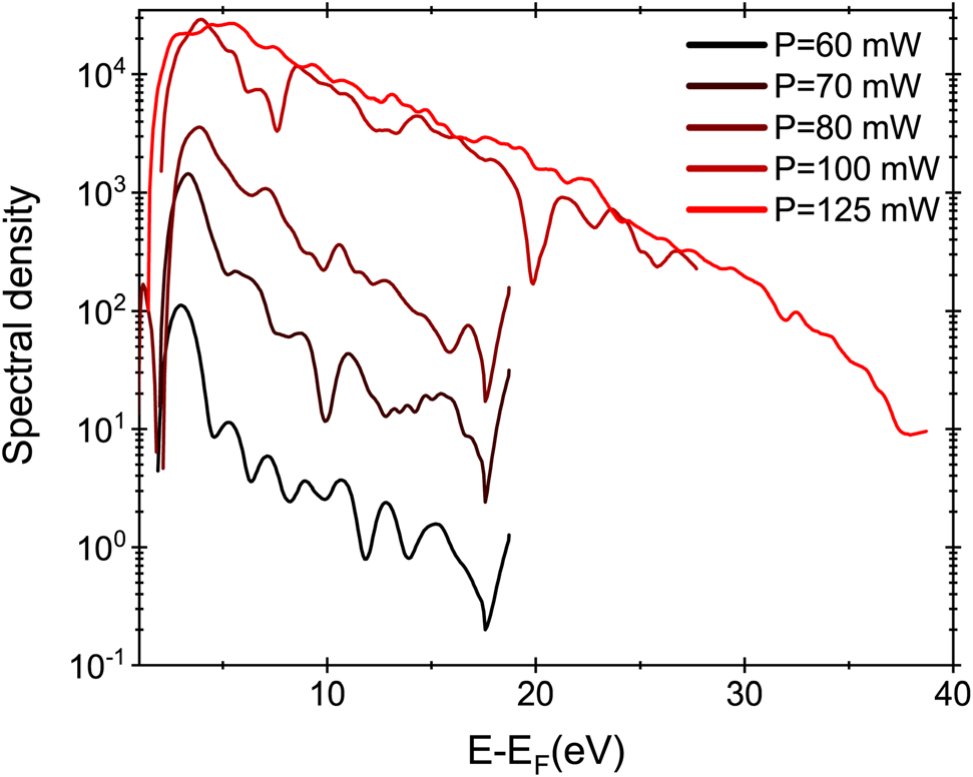
Energy spectra for *V* = 100 V under laser illumination varying the intensity from 12 GW cm^−2^ (red) to 25 GW cm^−2^.

The applied voltage was decreased to 100 V and the spectra were acquired by varying the laser intensity from 12 GW cm^−2^ to 25 GW cm^−2^ (*P* = 125 mW). By increasing the laser intensity, the position of the maximum of the energy peak shifts from 3 eV to 5.4 eV, indicating that the number of photons involved increases from 5 (for 60–70 mW) to 7 photons (for 80–100 mW). The ponderomotive energy at high laser intensity is about 1.5 eV closing the channel for 4, 5 and 6 multi-photons emission paths. Moreover, at high laser intensity, high energy electrons with *E* = *E*_F_ + 20 eV are also detected. The same spectra represented on semi-log scale (Fig. 5) show that we are able to detect electrons with energies up to =*E*_F_ + 35 eV. However, the energy plateau observed for strong field emission regime using mid-IR laser pulses is not reported in our analysis because we are just at the threshold of strong field phenomena.^34,35^ In fact the Keldysh parameter *γ* = *ω*/*ω*_t_ = 0.8, with 
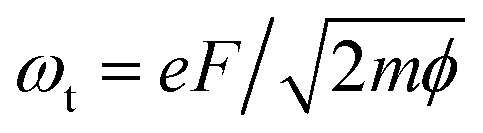
 and the optical field *F* given by eqn (3) for an enhancement factor of *ξ* = 11 for *I* = 25 GW cm^−2^. Strong field phenomena are reported for *γ* > 1. However, the laser intensity could not be increased further because the emission intensity would become unstable because of the migration of surface atoms.

At 100 V, for laser intensities lower than 25 GW cm^−2^, the emission pattern presents the four-fold symmetry, as reported in Fig. 4(e). However, at the laser intensity of 25 GW cm^−2^ the FEM pattern shows a strong emission from a broad and diffuse ring that joins the different (011) emission sites together. At high laser intensity, the emission follows the above threshold photoemission (ATP) (as shown in Fig. 3(c)) and it is less sensitive to the local work function and more sensitive to the local laser field enhancement. For higher laser intensities the emission becomes unstable. Due to the high repetition rate of the laser, at high laser intensities, the laser heats the field-emitter so much that the atoms diffuse at the surface changing the emission pattern. In addition, the current stability is reduced.

## 4 Conclusion

In conclusion, we show that LaB_6_ nanotips have high emission stability and strong brightness under the illumination by an ultra-fast illumination at 2.25 μm. We demonstrate better performances as compared to the metallic ultra-fast field-emitters under high amplitude and average power of laser radiation. Moreover, we show that the electron emission regime can be tuned by changing the applied extraction voltage and/or the laser power from the photo-field emission (PE) to the multiphoton photoemission regimes (MPP) or even to the above threshold photoemission (ATP). In the PE regime we reported a strong enhancement of the dc emission current under laser illumination. In the MPP regime we measured a long term stability and a high emission brightness. We show that the emission pattern changes from a single spot to 5 spots with a four-fold symmetry corresponding to the emission from the central (001) pole and the 4 (011) poles. At higher energy the FEM pattern evolves towards a ring shape pattern, keeping a high emission brightness. The energy spectrum of the emitted electrons strongly depends on the illumination conditions, so that in the ATP regime high energy electrons can be detected.

We proved the capability of these field emitters to work at high repetition rates (13 MHz), without being destroyed and without the appearance of thermal emission effects for laser intensity lower than 25 GW cm^−2^. This work showed that LaB_6_ needles are excellent candidates for building new high repetition-rate ultra-fast electron sources with high versatile properties.

## Conflicts of interest

There are no conflicts to declare.

## Supplementary Material

NA-005-D3NA00069A-s001
