# Supplementary material for: Bright and ultrafast electron point source made of LaB6 nanotip
Source: Nanoscale Adv. 2023 Mar 24;5(9):2462–9. doi: 10.1039/d3na00069a (PMC10153084; doi:10.1039/d3na00069a)
Supplement: NA-005-D3NA00069A-s001 [file NA-005-D3NA00069A-s001.pdf]

Electronic Supplementary Material (ESI) for Nanoscale Advances.
This journal is © The Royal Society of Chemistry 2023

Supporting Information: Bright and ultrafast electron point source made of LaB_6 nanotip.

O. Bhorade,[†] B. Deconihout ,[†] I. Blum,[†] S. Moldovan,[†] J. Houard,[†] A. Normand,[‡] K. Jagtap,[‡] M. More,[‡] and A. Vella[*,†]

†*Univ Rouen Normandie, INSA Rouen Normandie, CNRS, Groupe de Physique des Matériaux , Avenue de l'Université BP 12, 76801 Saint Etienne du Rouvray, France*

‡*epartment of Physics, Savitribai Phule Pune University, Pune, 411007, India*

E-mail: angela.vella@univ-rouen.fr

Phone: +33 (0)232 955168. Fax: +33 (0)232 955054

Supporting Information Available

Measure of electron current as a function of laser power and polarization angle

We measure the emitted current as function of the laser intensity at V=100V, in the MPP regime. The linear behavior reported on the log-log scale (see Fig. S1) can be fitted by the power low $I \propto P_{laser}^{n}$ with $n = 3.6 \pm 0.1$.

We measure the emitted current as function of the angle α between the electric field of the laser and the major axis of the field emitter, in the MPP regime (see Fig.S2). The fit by a sinusoidal function $I \propto (\cos(\alpha))^{2n}$ with $n = 5$ shows that in the MPP regime a mean number of 5 photons is involved in the emission process.

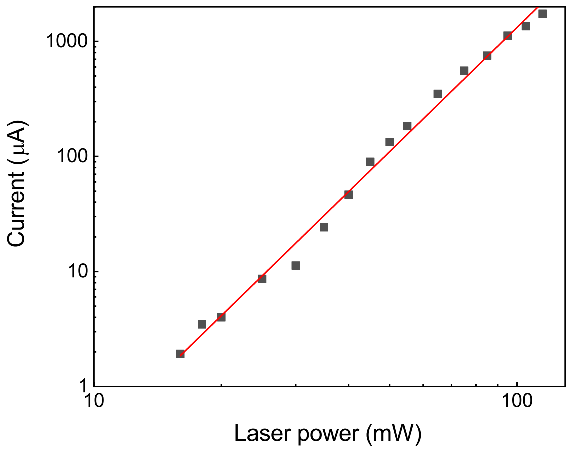

Figure S1: Electron current as a function of laser power (black square) at V=100V and fit (red line) using a power law: $I \propto P_{laser}^{n}$ with $n = 3.6 \pm 0.1$, indicating the MMP emission process.

Emission pattern and energy spectra in the MPP regime at low laser intensity

We report in Fig.S3 the FEM pattern at V=300V and I=5 GW/cm^2 and the energy spectra for each area indicated by a yellow circle. The main emission is from region R5 corresponding to the (001) pole. The energy spectra show a lower energy for electrons from region R5 due to the lower work function of (001) pole compared to (011) poles.

Brightness calculation

The Fig.S4 shows the set-up dimensions, the electron trajectories, the associated effective radius r_v and the semi-angle of divergence α. The m factor, known as the projection point factor, is calculated measuring on the screen the distance D between the central $< 001 >$ pole and the$< 110 >$ pole on a FIM image. Because for a cubic crystal the angular distance

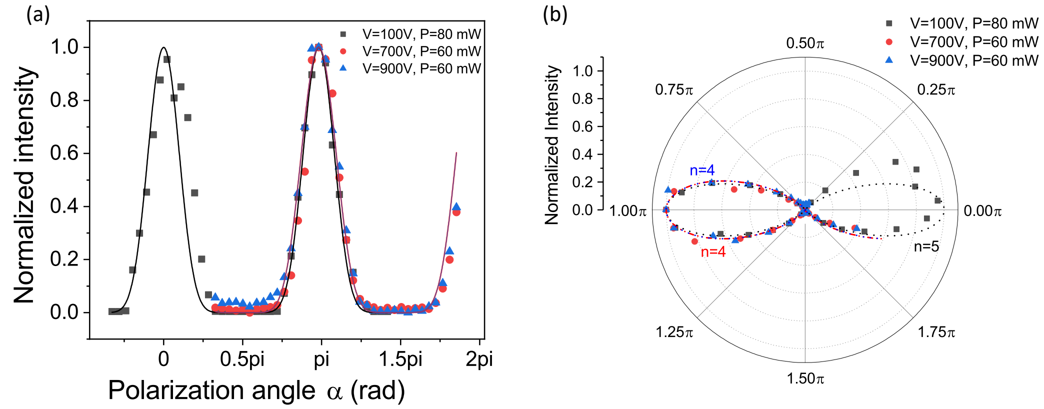

Figure S2: (a) Linear and (b) polar representation of emitted current as a function of the angle α between the electric field of the laser and the major axis of the field emitter.

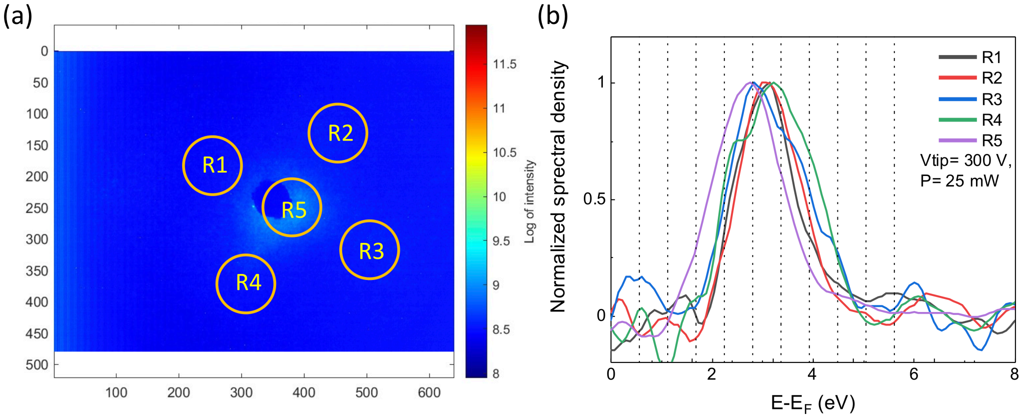

Figure S3: (a) FEM pattern at V=300V and I=5 GW/cm^2. (b) Energy spectra for each area indicated by a yellow circle in (a) at V=300V and I=5 GW/cm^2.

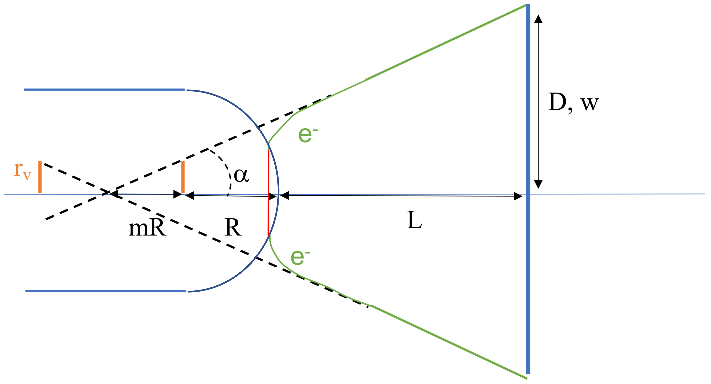

Figure S4: Electron trajectory diagram showing the set-up dimensions and the definition of the effective radius r_v for the emission area.

between these two poles is 45° we obtain:

$$m + 1 = \frac{45}{\arctan(D/L)}$$

with L the tip-screen distance. For $D = 3cm$ and $L = 6.5$ cm, $m = 0.8$.

The solid angle is calculated as: $\theta = 2\pi(1 - \cos(\alpha))$, with

$$\alpha = \arctan(w/L),$$

where $w = \frac{1}{2}$FWHM of the gaussian profile of the emission spot. The physical radius of the electron source is calculated as:

$$r_v = mR\tan(\alpha)$$

and the physical source size as: $S_v = \pi * r_v^2$. The brightness is:

$$B = \frac{I}{\pi r_v^2 \theta},$$

with I the emitted current. For static emission we measure current up to 10 nA, using a picoammeter between the high voltage power supply and the sample. However, due to the limitation of our detector, we can not register the associated FEM images.

Table 1: default

Voltage (V)	Power (mW)	Current (pA)	FWHM (mm)	α(rad)	θ (sr)	r_v(nm)	B×10^9 A/(m^2Sr^1)
				Sample 1			
660	0	0.5	4	0.03	0.003	0.246	0.88
800	0	6	7	0.05	0.009	0.430	1.13
400	40	0.25	7	0.05	0.009	0.430	0.047
				Sample 2			
465	0	0.1	4	0.03	. 0.003.	0.246	0.17
485	0	0.25	5	0.038	0.0046	0.307	0.18
565	0	5	6	0.046	0.0067	0.370	1.75

For lower current values, we measure the current on the phosphor screen I_{screen} and then we calculated the emitted current dividing I_{screen} by the detector gain-factor G. The gain-factor was measured at low emission currents, counting individual impacts on the screen. For an MCP voltage of 1550 V, $G = 10^7$. The values of current and the corresponding brightness are reported on the Table. For example, at a voltage of 565V we measured a current of 5pA, a semi-angle of divergence of 0.046 rad, an effective radius of 0.37 nm and a brightness of 1.75 $\times 10^9$ Am^{-2}Sr^{-1}. These value is lower than what was reported in the recent works on the emission from LaB_6, however, if we suppose that the solid angle of emission stays almost stable increasing the applied voltage from 500V to 850V and the emitted current from 5 pA to 10 nA, then we obtain a brightness of 10^{12} Am^{-2}Sr^{-1} close to the values reported in the literature.
